# *Yukmijihwang-tang* for the treatment of xerostomia in the elderly: study protocol for a randomized, double-blind, placebo-controlled, two-center trial

**DOI:** 10.1186/1745-6215-14-281

**Published:** 2013-09-03

**Authors:** Gajin Han, Jae-Woo Park, Seok-Jae Ko, Jihee Son, Jongki Seon, Juyeon Kim, Seulki Kim, Inkwon Yeo, Bongha Ryu, Jinsung Kim

**Affiliations:** 1 Department of Clinical Korean Medicine, Graduate School, Kyung Hee University, Seoul, Republic of Korea; 2 Department of Internal Medicine, College of Korean Medicine, Kyung Hee University, Seoul, Republic of Korea; 3 Department of Statistics, Sookmyung Women’s University, Seoul, Republic of Korea

**Keywords:** Xerostomia, *Yukmijihwang-tang*, *Yin-Deficiency*, Visual analogue scale

## Abstract

**Background:**

Xerostomia, a subjective sense of dry mouth, is not generally regarded a disease despite its high prevalence among the elderly, and therefore continues to impair affected patients’ quality of life. In traditional Korean medicine, ‘*Yin-Deficiency*’ has been implicated in the pathogenesis of xerostomia among the elderly. *Yukmijihwang-tang* is a famous herbal prescription used to relieve ‘*Yin-Deficiency*’, and reportedly has antioxidant effects; therefore, it is postulated that *Yukmijihwang-tang* can be used to treat xerostomia in the elderly. However, to our knowledge, no clinical trial has been conducted on the effects of *Yukmijihwang-tang* on xerostomia. Thus, we designed a randomized clinical trial to investigate the effects and safety of *Yukmijihwang-tang* on xerostomia in the elderly. In addition, we will clarify the aforementioned assumption that ‘*Yin-Deficiency*’ is the major cause of xerostomia in the elderly by identifying a correlation between xerostomia and ‘*Yin-Deficiency*’.

**Methods/Design:**

This randomized, double-blind, placebo-controlled trial will be carried out at two centers: Kyung Hee University Korean Medicine Hospital and Kyung Hee University Hospital at Gangdong. We will recruit 96 subjects aged 60-80 years who have experienced xerostomia for 3 months prior to participation. Subjects who present with score >40 on the visual analogue scale for xerostomia and unstimulated salivary flow rate under 0.3mL/min will be included and the randomization will be carried out by an independent statistician by using a random number creation program. The subjects and all researchers except the statistician will be blinded to the group assignment. *Yukmijihwang-tang* or placebo will be administered to each group for 8 weeks. The primary outcome is change in the scores for the visual analogue scale for xerostomia and the dry mouth symptom questionnaire from 0 to 8 weeks.

**Discussion:**

It will be assessed whether *Yukmijihwang-tang* can be used as a new herbal treatment for xerostomia in the elderly by demonstrating its therapeutic effects in a well-designed clinical trial.

**Trial registration:**

ClinicalTrials.gov Identifier: NCT01579877

## Background

Xerostomia, which is a subjective sense of oral dryness [1], is a fairly common condition among the elderly [2]. It is estimated that approximately 30% of the population aged 65 years and older have xerostomia [3], and several studies have reported a higher prevalence in the elderly than in the younger population [4-7].

The causes of xerostomia are long-term uses of xerostomia-inducing medications for chronic diseases and systemic diseases (for example, Sjögren’s syndrome, diabetes mellitus, Alzheimer’s disease, and Parkinson’s disease), head and neck radiation therapy, aging, heavy smoking, and stress [8]. In the elderly, the long-term use of medications and aging are regarded as major causes of xerostomia [9]. However, recent xerostomia studies mostly focused on Sjögren’s syndrome or postradiotherapy-induced xerostomia, which are related to salivary gland damage, and only a few studies have assessed xerostomia in elderly subjects who did not exhibit obvious structural damage of the salivary glands [10,11].

Xerostomia is not generally accepted as a disease despite its high prevalence in the elderly; therefore, this condition continues to impair the affected patient’s quality of life (QoL) [7,12]. For example, xerostomia in the elderly is known to cause dental problems and changes in chewing, swallowing, digestion, and taste [12]. Therefore, many xerostomia patients turn to solutions such as frequent water intake, sour foods, and alternative therapies (herbs or nutritional supplements) [13]. However, it is unclear whether these behaviors or therapies provide relief from xerostomia symptoms.

In traditional Korean medicine (TKM), ‘*Yin-Deficiency*’ is regarded as the main pathologic cause of xerostomia in the elderly [14]. Generally, the balance of *Yin* and *Yang* is important for maintaining health and treating diseases in TKM [14]. Particularly, *Yin* in ‘*Yin-Deficiency*’ refers to general body fluids, including blood, and *Jing* (mostly translated as ‘essence of body’) [15]. Therefore, ‘*Yin-Deficiency*’ is closely correlated with a shortage of body fluids, which results in various symptoms (dry mouth, dry eyes, or constipation) [14]. TKM has many herbal prescriptions for treating ‘*Yin-Deficiency*’ [10,16].

*Yukmijihwang-tang* (YMJ; *Liu-wei-di-huang-tang* in traditional Chinese medicine; *Lokumijio-to* in Kampo Medicine) is described in the ancient Chinese literature, ‘*Key to Therapeutics of Children’s Diseases*’ [17], and is composed of six herbs. It has long been used for enriching the fluid-humor of the body in TKM, and it can treat *Deficiency Fire Flaming Upward* due to ‘*Yin-Deficiency*’. Recently, many studies have described the pharmacological effects of YMJ, such as protection against renal ischemia/reperfusion in rats [18], memory enhancement in rats [19], and antioxidant and antidiabetic effects in early diabetic nephropathy patients [20]. In xerostomia-related aspects, YMJ is a famous herbal formula for body enrichment, which includes relieving ‘*Yin-Deficiency*’ [21], because of its demonstrated antioxidant and free radical scavenging activities in animal studies [18,22]. Thus, it is postulated that YMJ can improve xerostomia in the elderly by enriching the *Qi* of the body and tonifying *Yin*.

However, to our knowledge, no clinical trial has examined the effects of YMJ on xerostomia. Therefore, we designed a randomized clinical trial to assess the effects and safety of the use of YMJ on xerostomia in the elderly. To evaluate any changes in xerostomia symptoms, the visual analogue scale (VAS) for xerostomia and the salivary flow rate will be analyzed.

## Methods/Design

### Objectives

The aims of this study are to:

(1) obtain clinical evidence regarding the safety and effects of YMJ in elderly individuals with xerostomia;

(2) identify a correlation between xerostomia in the elderly and the ‘*Yin-deficiency*’ state.

### Hypothesis

We hypothesize that 8 weeks of YMJ administration will improve xerostomia in the elderly and will not induce severe side effects.

### Design

This is an academic research study that will be conducted as a randomized, placebo-controlled, double-blind, two-center trial at Kyung Hee University Korean Medicine Hospital and Kyung Hee University Hospital at Gangdong in Seoul, Korea.

This clinical trial will consist of an 8-week oral administration of YMJ with two visits at 4-week intervals and a 2-week follow-up period. Before enrollment, all subjects will go through a 7-day run-in period.

After randomization, all subjects will be divided into two groups: the YMJ and placebo groups. Each group will be provided with YMJ or a placebo for 8 weeks and the subjects will be required to take 3 g of YMJ or placebo three times a day. During the 8-week administration period, subjects will be prohibited from taking any kind of xerostomia-relieving drug such as pilocarpine or cevimeline. Primary and secondary outcomes will be measured at baseline and at 4 weeks, 8 weeks, and 10 weeks after randomization.

**Figure 1:**
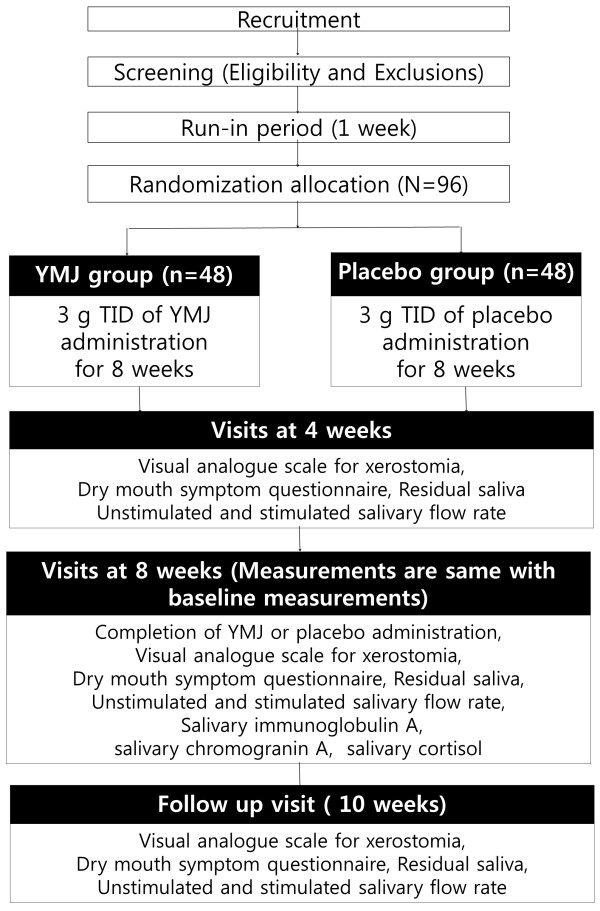
Flow chart of the study.

The flow of the entire trial is shown in Figure 1.

### Sample size calculation

The formula for estimating the sample size is as follows:

$$n_{t}=n_{c}=\left\{{\left(Z_{\alpha }{}_{/2}+Z_{\beta }\right)}^{2}\sigma^{2}\left(\lambda +1\right)/\lambda \right\}/{\left(\mu_{c}-\mu_{t}\right)}^{2}$$

A previous study demonstrated 1.54 points of improvement (*μ*_c_ - *μ*_t_= *Δ*) in the VAS for xerostomia after 4 weeks of treatment compared to that demonstrated after the placebo treatment [23]. The same study indicated a mean standard deviation (SD = *σ*) of 1.87. In our study, the ratio (*λ*) of the experimental group to the placebo group will be 1:1. With a power of 80% (1 - β) and a significance level of 5% (α), assuming Δ = 1.54 and σ = 1.87, a sample size of n_t_ = n_c_= 36 subjects per each group will be required (*n*_t_, number of YMJ group; *n*_c_, number of placebo group). Considering an assumed dropout rate of 25%, a total of 96 subjects will be needed. Although the intervention period in this study is different from that in previous similar study, we chose data from a previous study [23]. The rationale for choosing these data is the lack of studies or guidelines that used the same herbal prescription (YMJ) or the same outcome measure as this study.

On the other hand, to our knowledge, no clinical study has been conducted to assess the correlation between xerostomia in the elderly and the ‘*Yin-deficiency*’ state. Thus, the current trial is regarded as a pilot study to assess this correlation. This correlation will be assessed with a sample size of 96 subjects, which we believe is an appropriate sample size for evaluating this clinical correlation. The results will be shown by using descriptive statistical analysis.

### Inclusion and exclusion criteria

In general, the definition of elderly is used to describe people over the age of 60 years [24]; in accordance, we will recruit subjects who are between 60 and 80 years of age and have experienced xerostomia for 3 months prior to participation. We have restricted the subjects’ ages to below 80 years because individuals over this age are considered to have lower eligibility in this study on the basis of the rationale that the incidence of disease is increased as an individual ages.Therefore, we set an upper limit of 80 years on the subjects’ ages by a consensus among geriatric disease specialists.

The subjects should present with VAS scores >4 for xerostomia 2 weeks before the study period. In addition, the unstimulated salivary flow rates of the subjects should be under 0.3 mL/min. The subjects must agree that they will not receive xerostomia-related treatments for 3 months after initiation of the study.

Subjects who report the following conditions will be excluded:

(1) a history of treatments for autoimmune diseases like Sjögren’s syndrome, rheumatism, or lupus;

(2) a history of craniocervical radiation therapy, organ transplantation, severe psychiatric illness, or major depression;

(3) the use of any other herbal prescriptions or nutritional supplements at the screening phase;

(4) the use of xerostomia-relieving medications (pilocarpine and cevimeline) or therapies for xerostomia (mouthwash, gum, and toothpaste for xerostomia).

### Recruitment

We will carry out open recruitment by placing advertisements, and the decision to participate will be made by the subject.

Colored fliers, brochures, and banners will be placed inside of both hospitals. Furthermore, we will place advertisements in the newspaper and on the homepage of the hospitals and the boards of senior welfare centers around the hospitals.

### Randomization

The staff of Hanpoong Pharm & Food Co., Ltd. will first place a numbered label on each YMJ and placebo according to the randomization program results provided by the statistician, and the YMJ and placebo will subsequently be distributed to each clinical center. The randomization result table will remain sealed by the statistician.

After the subject has passed the screening phase, an independent clinical research coordinator (CRC) will control randomization. First, the randomization form containing the basic information of the subject who has passed the screening phase will be transmitted in facsimile to an independent statistician. The randomization number in the randomization form will be left blank before the statistician receives the form. The statistician will make a decision about the randomization number based on the allocation sequence that has been generated by a random number creation program in advance, and the statistician will return the randomization form filled in with the established number (specified ID number) to the CRC. Then, the clinical pharmacist will distribute the corresponding numbered YMJ or placebo to the subject.

The randomization allocation ratio to the sites will be 1:1. The CRC will provide the specified ID number to the investigators. The authorized contract research organization (CRO) will guarantee that all procedures are performed correctly.

### Blinding

The subjects will be blinded to the treatment that they receive. Additionally, the investigator, CRC, and clinical pharmacist will be blinded to the randomization. Only the independent statistician will be involved in the randomization, and the entire procedure will be audited by the authorized CRO (DreamCIS, Seoul, Korea).

### Intervention

#### YMJ

YMJ has long been used in TKM to treat symptoms like dry mouth or throat, emaciation, decreased urine, an irritable rash on the palms and soles, insomnia, and tidal fever caused by ‘*Yin-deficiency*’ [14]. It is composed of six herbs: *Rehmannia glutinosa*, *Cornus officinalis*, *Dioscorea batatas*, *Paeonia suffruticosa*, *Poria cocos*, and *Alisma orientale*[25].

The YMJ that will be used in this trial is a brown, bitter, granular, herbal extract (Hexalong granule®, Hanpoong Pharm & Food Co., Ltd., Jeonju, Korea) produced according to the Korean Good Manufacturing Practice guidelines. Hexalong granule® is permitted and regulated by the Korean Food & Drug Administration. Every 3 g of Hexalong granule (water-extracted YMJ) includes *Rehmannia glutinosa* (2 g), *Cornus officinalis* (1 g), *Dioscorea batatas* (1 g), *Paeonia suffruticosa* (1 g), *Poria cocos* (1 g), and *Alisma orientale* (1 g) as raw materials.

All herbs will be obtained from qualified suppliers in Korea, and water-extracted YMJ granules will be sealed in opaque aluminum bags.

We will administer YMJ to the subjects at a dose of 3 g according to the specialty publication of the standard guidelines of the herbal prescription administration [26].

#### Placebo

Presently, a standard treatment for xerostomia does not exist. Thus, the placebo YMJ that will be used in this study does not have active components. The placebo YMJ was made with cornstarch powder that has the same color and taste as YMJ by Hanpoong Pharm & Food Co., Ltd. by using their standard method of placebo manufacturing according to the Korean Good Manufacturing Practice guidelines. The placebo YMJ will be packed identically in opaque aluminum bags with the same labeling form as real YMJ.

#### Distribution and administration

An independent pharmacist will distribute the experimental agent packages to the subjects in a separate room and the CRC will check to ensure that the distribution is correct. The subjects will be instructed to dissolve the YMJ or placebo granules from each package in water and take them 2 h after each meal. Voucher specimens of YMJ will be retained at the research laboratory of the manufacturer.

The investigator will monitor compliance with the study treatments by carrying out a telephone conference once a week between on-site visits. During the telephone conference, the investigator will inquire how many study agents the pharmacist has administered and will record the number on a case report form (CRF). If a subject does not take all of the YMJ or placebo, they will be asked to return the unused portion to the CRC at the next visit. The CRC and clinical pharmacist will double-check and document the amount of the returned portions. Finally, clinical research associates (CRAs) will check compliance with the study agents at the end of the study.

YMJ and its placebo will be kept and managed by clinical pharmacists who are independent of the investigators. The clinical pharmacists will record the date and the number of study agents during distribution and return.

### Outcome measurement

#### Primary outcome

VAS for xerostomia was chosen as a primary outcome to evaluate the subject’s improvement regarding discomfort due to xerostomia (ranging from 0 mm as no xerostomia to 100 mm as the most severe xerostomia). The measurements of VAS will be carried out at screening, baseline, at 4 weeks, and 8 weeks during YMJ administration, and at the end of study (10 weeks after baseline). The change of VAS scores before and after intervention (from week 0 to week 8) will be compared between the YMJ and placebo groups.

#### Secondary outcomes

##### Dry mouth symptom questionnaire

The dry mouth symptom questionnaire (DMSQ) [27] is composed of six questions to evaluate subjective oral dryness by using the VAS and four questions to evaluate behavior to avoid oral dryness (using the Likert scale). The measurements will be carried out at baseline and at 4 weeks, 8 weeks, and 10 weeks.

##### Unstimulated and stimulated salivary flow rate

The unstimulated (USFR) and stimulated salivary flow rate (SSFR) are secondary outcomes that will be used to evaluate the effects of YMJ. The overall salivary flow rate will be determined by the draining method [28]. Briefly, the subjects will be asked to rest for 10 min before clearing the mouth by swallowing all saliva within the oral cavity. Then, they will be instructed to place their tongue above a graduated cylinder (marked to the nearest 0.1 mL) for 10 min, and the drooled saliva will be collected. After 10 min of collection, the volume of the collected saliva will be measured. According to the criteria proposed by Ericsson and Hardwick [29], hyposalivation will be defined as an USFR <0.1 mL/min.

Next, the subjects will be asked to chew a paraffin film (Parafilm: Pechiney Plastic Packaging Company, Chicago, IL, USA) and the saliva that is induced by chewing will be collected in a graduated cylinder. After 5 min, the volume of the saliva will be measured. These measurements will be carried out at screening, baseline, 4 weeks, 8weeks, and 10 weeks.

##### Residual saliva

The volume of residual saliva in the subject’s mouth will be measured to examine the effect of YMJ on the change in intra-oral saliva. The investigator will check the volume of residual saliva on the subject’s tongue and bucca by using the Moisture Checker for Mucus (MCM: Life Co, Ltd, Tokyo, Japan). This measurement will be carried out at baseline, 4 weeks, 8 weeks, and 10 weeks.

##### Salivary immunoglobulin A, salivary chromogranin A, and salivary cortisol

Salivary immunoglobulin (Ig) A, chromogranin A, and cortisol are well-established biologic markers of stress in the human body and are associated with salivary gland secretion [30-34]. These measurements will be carried out at baseline and at 8 weeks.

In our study, the saliva sample will be collected between 09:00 and 10:00, and the salivary cortisol concentration will be determined by using the ER HS SALIVARY CORTISOL kit (Salimetrics, State College, PA, USA). The salivary Ig A will be examined by using the Salivary Secretory Ig A indirect enzyme Immunoassay Kit (Salimetrics, State College, PA, USA). Salivary chromogranin A will be assessed by using the Chromogranin A (Human) EIA Kit (Yanaihara, Japan). The analysis will be conducted by using a microplate reader (Molecular Devices, Sunnyvale, CA, USA), according to the manufacturer’s instructions.

##### ‘Yin-deficiency’ Questionnaire 1

The development and validation of the ‘*Yin-deficiency*’ questionnaire 1 (Yin-DQ1) was described in a previous study [14]. This questionnaire consists of 10 VAS-related items: an irritable rash on the palms or soles, flushing of the zygomatic area in the afternoon, tidal fever, night sweats, emaciation or weight loss, dry mouth and/or throat, dizziness, constipation, decreased urine with yellowish color, and insomnia. The subjects will take this questionnaire at baseline and 8 weeks.

#### Safety

We will perform the following tests on all subjects at screening and after completion of YMJ administration: complete blood cell count; levels of aspartate aminotransferase/alanine aminotransferase, gamma-glutamyltranspeptidase, blood urea nitrogen, and creatinine; and erythrocyte sedimentation rate. These blood tests will help us to exclude subjects who have serious diseases and abnormal liver, heart, kidney, or other organ function. Throughout the present study, we will also check whether the 8-week administration of YMJ in xerostomia patients is safe by using these tests or CRF documentations.

In addition, at each visit, investigators will ask the subjects whether there are any adverse events (AEs) during the study period. If there is any AE, the investigator will provide the appropriate treatment to the subject immediately and record AEs in the dedicated document of the CRF including its severity and causality with the experimental agent. These AEs occurred during the entire study will be reviewed and monitored by independent CRA.

In the case of serious adverse events (SAEs), the investigator will offer the corresponding treatment to the subject immediately and report to IRB within 24 h from the time of recognition. If necessary, blind will be broken by adequate procedure and the documented procedure will be kept in the investigator study file.

#### Quality control

To retain the accuracy and quality of the clinical trial, DreamCIS, an established CRO located in Seoul, Korea, will audit the study. In addition, regular monitoring will be implemented by the CRA to ensure that the clinical trial is proceeding according to the protocol by checking trial master files, informed consent forms, the CRF, adverse events, and compliance with study agents.

#### Statistical analysis

An independent statistician will perform a blind statistical analysis using SPSS 16.0 (SPSS Inc., Chicago, IL, USA). All data will be presented as means and SDs, and all analyses will be based on the intention-to-treat principle.

The analysis strategies in the present study are as follows.

First, we will use the ANCOVA to compare the changes in VAS scores (primary outcome) to evaluate the effects of YMJ and the placebo from the beginning (0 week) to the end (8 weeks) of the study period. Second, the secondary outcomes (DMSQ scores, USFR, SSFR, volume of residual saliva, salivary Ig A, chromogranin A, cortisol, and ‘*Yin-deficiency*’ score) will be analyzed in the same manner as the primary outcome. Finally, correlation between subjective (VAS and DMSQ) and objective xerostomia-related variables (USFR, SSFR, and residual saliva) will be analyzed by Pearson’s correlation coefficients. Also, we will find out how xerostomia-related variables correlate with ‘*Yin-deficiency*’ score or stress-related variables (salivary Ig A, chromogranin A, and cortisol), respectively, using Pearson’s correlation coefficients. In addition, we will check the AEs in YMJ and placebo group each and describe them using the descriptive statistics.

### Ethical approval and registration

This study will be carried out in accordance with the standards of the International Committee on Harmonization on Good Clinical Practice and the revised version of the Declaration of Helsinki. The protocol of the trial has been approved by two ethics committees: the Institutional Review Boards (IRBs) of both the Oriental Hospital at Kyung Hee University Medical Center and the Kyung Hee University Hospital at Gangdong. The permission numbers are KOMCIRB 2011-28 for the Oriental Hospital at Kyung Hee University Medical Center and KHNMC-OH-IRB 2011-016 for the Kyung Hee University Hospital at Gangdong.

The process of obtaining consent will be carried out by the principle of informed consent, which consists of information, decisional capacity, and voluntarism. Prior to screening, written informed consent approved by the IRB will be obtained from all participants who present at a clinic in response to an advertisement. The investigator will explain the study by using non-scientific language before the subjects read the terms of consent. Subjects will be given adequate time to decide whether they wish to participate before signing the consent form. After this process, if the subject spontaneously wishes to participate in the study, they will sign the consent form.

This trial is registered with the ClinicalTrials.gov protocol registration system, ID NCT01579877.

## Discussion

This clinical trial, which will be conducted as one of the nationwide projects to provide scientific evidence for TKM, belongs to the 2011 Traditional Korean Medicine R&D projects funded by the Ministry for Health & Welfare & Family Affairs, Republic of Korea.

The present study emphasizes the significance of treatment for xerostomia in the elderly, which has been neglected clinically up to this point, and can play an important role in giving prominence to treatment for this condition. We anticipate that this study will reveal that a herbal prescription and a typical TKM treatment method, YMJ, can be an alternative treatment for xerostomia in the elderly, a condition that presently has no suitable remedy.

In general, herbal medicine is a key tool of TKM treatment and is known to have a great curative power for many diseases [35,36]. YMJ, which is one of the most frequently used herbal formulas in Korea, is regarded to be safe and to have a beneficial effect on xerostomia in the elderly [21]. However, to our knowledge, no randomized controlled trials have shown the effectiveness of YMJ for xerostomia. Therefore, the current study was designed to obtain objective clinical evidence on the effects and safety of YMJ for the treatment of xerostomia in the elderly. If the effects of YMJ are verified in the present study, it could be used as a new alternative treatment option by elderly individuals with xerostomia, which is highly prevalent and without established standard therapies in conventional medicine.

The current study has three potential strengths. First, it is unique because the subjects of this study will have general xerostomia without the structural damage to the salivary glands that occurs with autoimmune disease or a history of radiation. Second, the ‘*Yin-deficiency*’ questionnaire, an indigenous pattern identification tool in TKM, will be applied to diagnose the subjects’ xerostomia according to TKM theory. If a high correlation is found between xerostomia in the elderly and ‘*Yin-deficiency*’, this will provide reasonable evidence for prescribing YMJ for xerostomia in the elderly. Third, we will determine whether YMJ can change the subjective feeling of dry mouth as well as the salivary flow rate. Although the relationship between a subjective feeling of dry mouth and the salivary flow rate is controversial [8,9,37], the change in these factors in association with YMJ treatment could be compared in this study.

## Trial status

This study is currently recruiting patients.

## Abbreviations

AE: Adverse event; CRA: Clinical research associate; CRC: Clinical research coordinator; CRF: Case report form; CRO: Contract research organization; DMSQ: Dry mouth symptom questionnaire; Ig A: Immunoglobulin A; IRB: Institutional review board; QoL: Quality of life; SAE: Serious adverse event; SSFR: Stimulated salivary flow rate; TKM: Traditional Korean medicine; USFR: Unstimulated salivary flow rate; VAS: Visual analogue scale; YMJ: *Yukmijihwang-tang.*

## Competing interests

The authors declare that they have no competing interests.

## Authors’ contributions

JSK, JWP, and BHR contributed to securing funding for the project and to the study design. GJH, SJK, JHS, JKS, JYK, and SKK participated in the design of the trial. JSK, JWP, and GJH drafted the protocol and wrote the final manuscript. JWP and IKY were responsible for the statistical design of the trial. All authors read and approved the final manuscript.
